# In Situ Simulation to Promote Residents as Resuscitation Leaders

**DOI:** 10.7759/cureus.14449

**Published:** 2021-04-13

**Authors:** Andrew Kalnow, Alex Davis, Zach Hampton, Brad D Gable

**Affiliations:** 1 Emergency Medicine, OhioHealth Doctors Hospital, Columbus, USA; 2 Emergency Medicine, Ohio University Heritage College of Osteopathic Medicine, Athens, USA; 3 OhioHealth Learning, Riverside Methodist Hospital, Columbus, USA

**Keywords:** simulation, in situ simulation, medical education, resident education, emergency medicine

## Abstract

Objectives

Our study sought to assess whether perceptions of residents as resuscitation team leaders could be improved by using emergency department (ED) in situ simulations involving ED staff. Secondarily, we monitored changes indicated in overall resuscitation team dynamics.

Methods

We conducted a prospective experimental study over the 2018-2019 academic year. Data were collected at a community-based ED with an emergency medicine residency program. Prior to starting the education, all ED staff including residents, attending physicians nurses and techs completed a survey of their perceptions of team performance and leadership during medical resuscitations. Throughout the year, residents and ED staff members were exposed to various in situ simulation scenarios. A follow-up survey was administered to reassess resident and ED staff perceptions of team dynamics and, specifically, residents as patient care team leaders. A relational coordination survey analysis was performed, dichotomized by professional domain.

Results

A total of 20 participants completed surveys before and after the in situ simulations, covering the professional domains with matched pre-simulation and post-simulation data showing significant improvement in communication and team dynamics for residents (p = 0.029) and other ED staff in medical resuscitations. Using residents as the team leaders for the simulation improved perceived leadership during resuscitation (p = 0.006).

Conclusions

Our study suggests that in situ simulation within the ED leads to improved team dynamics and defined roles while emphasizing the resident as a resuscitation leader.

## Introduction

In situ simulation in the emergency department (ED) involves moving patient scenarios out of the simulation lab and into the ED, where patient care teams can interact in a more realistic environment than the lab setting. In situ simulations, particularly in the setting of trauma teams, can improve a wide variety of skills including problem identification, decision-making, time management, and confidence [1-3]. Multidisciplinary in situ simulation improve resident education [4,5] and can improve ED protocols through the identification of errors and latent safety threats [6,7]. These skills also improve in non-physician ED team members, including nurses, though it is not clear how long these skills are maintained [1]. To our knowledge, the effect of in situ simulation on improving resuscitation communication and leadership in emergency medicine residents has not been previously assessed. Our study sought to assess whether perceptions of residents as resuscitation team leaders could be improved by using ED in situ simulations involving ED staff. Secondarily, this study evaluated the overall impact of the training sessions on team communication and dynamics.

## Materials and methods

We conducted a prospective experimental study over the 2018-2019 academic year. Data were collected at a community-based ED with an emergency medicine residency consisting of 32 postgraduate years (PGYs) 1-4 residents plus approximately 80 ED team members, including attending physicians, nurses, and support staff. The ED serves >70,000 patients annually and is receiving chest pain and primary stroke center with diverse specialty care available. Institutional Review Board approval was obtained and was approved under the exempt category one approval. Residents, attending physicians, nurses, techs, and patient care assistants, completed a voluntary pre-simulation survey assessment of their perceptions on team performance and leadership during medical resuscitations. The survey focused on the following professional domains: patient care, ED team (medical and trauma), leadership, and simulation. After one academic year of monthly in situ simulation sessions, a post-simulation survey assessment identical to the pre-simulation survey was administered to reassess physician and ED staff perceptions of team dynamics with a specific emphasis on residents as patient care team leaders. Surveys were completed anonymously online by participants without obligation and tracked only by professional domain. 

At the beginning of each simulated case, participants were given a scenario with assigned roles. However, all participants were blinded to intended outcomes within the study. Data collection was completed via REDCap [8,9], the institutional data collection tool, and reported in a Microsoft Excel spreadsheet without participant identifiers [5,6]. As the year progressed, residents and ED staff members were exposed at random to high-level critical resuscitations, stressing the need for effective communication, utilization of existing processes, leadership, and crisis resource management. Both medical and trauma scenarios were administered including decompensated heart failure, precipitous delivery with newborn resuscitation, trauma patient with a pelvic fracture and subdural hemorrhage, and a trauma patient with a stab wound to the neck (Table 1). Emphasis was placed on having the senior resident (PGY 3-4) perform as a team leader within the resuscitation; attending physicians were encouraged to observe but not directly participate in the simulated resuscitations.

**Table 1:** Summary of Simulation Cases Used STEMI: S-T segment elevation myocardial infarction, CHF: congestive heart failure, NOAC: novel oral anticoagulant, ICP: increased intracranial pressure, ICH: intracranial hemorrhage.

Case	Scenario	Critical Actions
Precipitous delivery and newborn resuscitation	19yo female presenting as a precipitous delivery with a nuchal cord delivery. Newborn requires resuscitation following delivery.	Recognize and address a precipitous delivery with nuchal cord presentation and subsequent need for neonatal resuscitation.
Cardiogenic shock with STEMI	55yo male presenting with a decompensated CHF exacerbation that progresses to cardiogenic shock secondary to a STEMI	Recognize and treat a dynamic case with decompensating heart failure and STEMI including the need for resuscitation prior to disposition to interventional cardiology.
Stab wound to the neck	21yo male with a stab wound to the neck presenting with concerns for a zone 2 neck injury and neurogenic shock	Identify the need for a significant neck injury needing both cervical spine precautions and immediate airway management.
Fall from a horse with pelvic fracture and subdural hematoma	32yo female bucked off her horse presents altered with concerns for a head injury and pelvic fracture.	Identify and address multi-trauma concerns including hypotension due to both pelvic fracture and head injury.
Fall on NOAC with a head injury	81yo male fall downstairs. The patient on NOAC shows signs of ICP/ICH.	Recognize patient critical condition, address anticoagulation reversal and airway management.

A relational coordination survey (RCS) analysis, dichotomized by profession domain, was performed. The RCS was chosen as a means to evaluate teamwork, communication, and collaboration for a team with defined roles [10]. Questions involving the perception of resuscitation focused on team communication, goals, problem-solving, and understanding of workflow based on professional domain. The maximum score for this section was 35. The scoring of questions specifically regarding staff perception of leadership during a resuscitation event had a maximum value of 45. By using an RCS, we sought to better understand the relationships between the various ED roles in a critically ill patient resuscitation [11].

## Results

A total of 63 participants completed the pre-simulation survey of whom 20 matched participants completed a post-simulation survey (response rate, 32%). When matched by professional domain, pre-simulation and post-simulation data showed significant improvement in communication and team dynamics (Table 2). Specifically, the groups that noted improvement in communication and team dynamics were residents (p = 0.029), nursing (p = 0.030), and support staff (p = 0.021). No change in perceived communication and team dynamics was observed within the attending domain. In addition, the residents’ perceived leadership during critical resuscitation did show improvement (p = 0.006).

**Table 2:** Relational Coordination Survey Results by Professional Domain (n=20) ^a^Maximum score: 35, ^b^Maximum score: 45

	Pre-simulation Composite Score	Post-simulation Composite Score	P-value
Medical resuscitation^a^	Mean ± SD	Mean ± SD	
Resident	28.8 ± 3.6	31.2 ± 2.5	0.029
Attending	29.0 ± 3.4	30.6 ± 3.4	0.150
Nursing	27.0 ± 3.1	29.5 ± 3.1	0.030
Support staff	24.6 ± 4.2	26.8 ± 4.1	0.021
Leadership^b^	36.9 ± 5.3	41.2 ± 4.3	0.006

## Discussion

While in situ simulation topics can vary widely in different clinical settings, many serve to both focus on the resuscitation of a critically ill patient as well as team dynamics. These team dynamics include leadership, communication, and crisis resource management. In situ simulations may be of particular benefit for rising junior and senior-level residents not only for improving their leadership and communication skills but also promoting these residents as leaders with the team members they work with. For a senior resident, successfully leading a critically ill patient resuscitation is the pinnacle of training. However, based on prior feedback, the progression into a leadership role can be hindered by the tendency of ED staff to gravitate to the attending physician for guidance and leadership.

In our study, having the senior resident serve as the leader of the resuscitation was intended to give structure to the room during stress-provoking situations. The lack of change noted in the attending category is likely due to the attending physicians observing the scenarios, but not included as active participants in the simulated resuscitations. Also, given that nurses and support staff already look to the attending physician for leadership and guidance, an in situ simulation would not evoke a change from baseline.

Our results support the use of in situ simulation in the ED as a tool for resident leadership development. Given the critical nature of these simulations, there was more perceived confidence in resident leaders after the simulation and thus contributed to the significant improvement in communication and leadership scores.

LIMITATIONS

Our study has several limitations. The innate nature of bias is difficult to avoid in a simulation setting; however, we attempted to mitigate reporting bias by ensuring participating residents and ED staff did not know the study variables. Additionally, participants were encouraged to focus on resuscitation management with specific attention given to communication during the resuscitation. Also, this was a small study within a single community ED, which limits the generalizability of our findings. Approximately 32% of the ED staff, including residents, returned surveys that could be matched for analysis. The 32% response rate was in part due to staff turnover at 12 months, as well as the survey being voluntary and complex. As each case had its own set of challenges, we could not discern if each case scenario equally impacted team dynamics and resident leadership. However, we maintain that the act of participating together within a challenging case affects team dynamics. Participants in each case were not standardized nor were they randomized. While one resident from each PGY was included in each simulation, nurses, and support staff participated at random based on availability. The effort, time, and resources to make these scenarios a reality may limit the replication of this study at a facility with different resources. Moving forward, generalizability can be improved by involving hospital systems rather than a single community ED. Academic centers would likely benefit by repeating this study to elicit changes in resident perceptions within various domains, as there may be a difference in the culture of each program or department. Ultimately, the goal is to determine if the in situ simulation can change patient outcomes, including decreased morbidity and mortality through better medical management or communication.

## Conclusions

Our goal was to assess whether perceptions of residents as resuscitation team leaders could be improved by using ED in situ simulations involving ED staff. Communication, team dynamics, and leadership can be improved through in situ simulations in the ED. Our findings should empower those looking to improve the curriculum of resident education to include in situ simulation within their own EDs.

## Appendices

In Situ Simulation Pre and Post-Survey

Section 1: Emergency Department Team (Relational Coordination Survey)

Responses based on:

              1                     2                        3                        4                      5

       Disagree                        Neither Agree nor Disagree                 Agree

During critical MEDICAL resuscitation how much do you feel that people in each of the groups

1)    Communicate with you as frequently as needed

Residents/Fellows

Attendings

Nurses

Support Staff (Medics/Techs/UC)

2)    Communicate with you in a timely manner

Residents/Fellows

Attendings

Nurses

Support Staff (Medics/Techs/UC)

3)    Communicate with you accurately (closed-loop communication)

Residents

Fellows

Attendings

Nurses

Support Staff (Medics/Techs/UC)

4)    Work with you to solve task problems

Residents/Fellows

Attendings

Nurses

Support Staff (Medics/Techs/UC)

5)    Share your goals

Residents/Fellows

Attendings

Nurses

Support Staff (Medics/Techs/UC)

6)    Understand your work and role

Residents/Fellows

Attendings

Nurses

Support Staff (Medics/Techs/UC)

7)    Respect your work

Residents/Fellows

Attendings

Nurses

Support Staff (Medics/Techs/UC)

During critical TRUMA resuscitation, how much do you feel that people in each of the groups

1)    Communicate with you as frequently as needed

Residents/Fellows

Attendings

Nurses

Support Staff (Medics/Techs/UC)

2)    Communicate with you in a timely manner

Residents/Fellows

Attendings

Nurses

Support Staff (Medics/Techs/UC)

3)    Communicate with you accurately (closed loop communication)

Residents

Fellows

Attendings

Nurses

Support Staff (Medics/Techs/UC)

4)    Work with you to solve you to solve task problems

Residents/Fellows

Attendings

Nurses

Support Staff (Medics/Techs/UC)

5)    Share your goals

Residents/Fellows

Attendings

Nurses

Support Staff (Medics/Techs/UC)

6)    Understand your work and role

Residents/Fellows

Attendings

Nurses

Support Staff (Medics/Techs/UC)

7)    Respect your work

Residents/Fellows

Attendings

Nurses

Support Staff (Medics/Techs/UC)

Section 2: Leadership

Please indicate your agreement with the following statements about leadership. Team leaders (resident and/or attending leading the resuscitation) ensure:

      1                    2                       3                        4                         5

Disagree                        Neither Agree nor Disagree                 Agree

1)    Their team has an engaging direction that is clear, simple, and specifies ends to be achieved rather than means.

2)    All team members are engaged and included in decision-making appropriately

3)    Their team reviews and reflects on their performance to identify opportunities for improvement

4)    There is clarity about the team’s membership

5)    Team members agree on measurable team objectives

6)    Tasks are delegated to team members appropriately

7)    There are constructive debates about how to provide and improve high-quality patient care

8)    Their team has adequate resources for the tasks they are assigned

9)    Their team is rewarded both as a group and individually for their work

10) Team members DO NOT have a role in team leadership
